# Exercise habits and C-reactive protein may predict development of spinal immobility in patients with ankylosing spondylitis

**DOI:** 10.1007/s10067-018-4195-y

**Published:** 2018-07-18

**Authors:** Björn Sundström, Lotta Ljung, Solveig Wållberg-Jonsson

**Affiliations:** 1 Department of Public Health and Clinical Medicine, Rheumatology, Umeå University, SE-901 87 Umeå, Sweden; 2 Clinical Epidemiology Section, Department of Medicine Solna, Karolinska Institutet, Stockholm, Sweden

**Keywords:** Ankylosing spondylitis, Biomarkers, Exercise, Physical activity

## Abstract

To assess predictors for spinal immobility in a long-term clinical study of patients with AS, data from annual clinical measurements of spinal mobility in 54 patients (41 men, mean of age at end of follow-up 54.7 years) with ankylosing spondylitis were co-analysed with data regarding lifestyle factors as well as laboratory measurements from a previous cross-sectional study. Spinal immobility was graded on the basis of recently published age-, sex- and length-specific reference intervals. Exercise habits and high-sensitivity C-reactive protein (hsCRP) were independently associated with the development of subnormal spinal immobility (*p* = 0.019 and *p* = 0.021). In multiple regression models, approximately 25% of the spinal immobility could be attributed to disease duration (*p* ≤ 0.011), levels of hsCRP (*p* ≤ 0.004) and exercise in leisure time (*p* ≤ 0.019). The mean concentration of hsCRP was 4.2 mg/L (range 0.2–8.4 mg/L) in the study cohort. Bath Ankylosing Spondylitis Disease Activity Index (BASDAI), erythrocyte sedimentation rate (ESR) and physical activity at work were not associated with spinal immobility. The results indicate that exercise habits may have an impact in preventing the development of spinal immobility in AS independently of disease duration and inflammation. This corresponds well with the accumulated knowledge from long-term clinical experience among rheumatologists, health professionals and patients. Consequently, exercise should remain an important part of the non-pharmacological treatment and self-care for patients with AS. Furthermore, modest inflammatory activity, measured as a slightly elevated hsCRP concentration, appears to affect subsequent spinal immobility in AS.

## Introduction

The decline of spinal mobility is a hallmark of ankylosing spondylitis (AS) [1]. In the early stages of the AS disease, spinal immobility can usually be related to inflammatory pain, but during the course of the disease, tissue calcification and bone ankylosis of the spine becomes increasingly important [2, 3]. To date, factors affecting the decline of spinal mobility and the ossification of spinal ligaments are poorly understood and it is complicated by the fact that spinal mobility decreases with age. However, recently published reference intervals (RI) for spinal mobility measurements in healthy individuals offer new possibilities to assess impairment during the course of the AS disease [4]. Identification of modifiable risk factors, as well as markers, for spinal immobility in AS would allow individualised preventive efforts.

Our aim in the present study was to determine whether modifiable risk factors and disease activity measurements among patients with well-established disease can be used to predict spinal immobility in a population of patients with AS in a long-term clinical follow-up.

## Materials and methods

### Setting and study population

Since the 1980s, all patients in the county of Västerbotten, northern Sweden, with a verified diagnosis of AS, have been offered treatment and a regular assessment, including spinal mobility measurement, at the Department of Rheumatology at Umeå University Hospital. The spinal measurements are performed annually by trained physiotherapists using a standardised protocol, although intervals between can be prolonged for patients with a slow disease progression. The study cohort in this follow-up study comprised 66 patients with a validated diagnosis of AS according to the modified New York criteria [5] who previously had participated in a cross-sectional study performed in 2008 [6]. The data, collected in 2008, comprised information of education level, social status, smoking history, dietary habits and pharmacological treatment, retrieved from questionnaires, as well as data from the Swedish version of the Bath Ankylosing Spondylitis Disease Activity Index (BASDAI) [7] and Bath Ankylosing Spondylitis Functional Index (BASFI) [8] were retrieved. Physical activity at work and exercise habits were assessed by asking the patients to choose statements that best resembled their habits and occupation. The questions and statements are described in detail previously [9]. In short, physical activity at work was assessed on a 4-point scale graded from mostly sitting with desktop work, to physically demanding work such as farming or construction work. Physical activity during leisure time was assessed on a 5-point scale ranging from no activity at all to strenuous exercise several times a week. In the cross-sectional study in 2008, weight and height were measured, and laboratory analysis performed for complete blood count, cholesterol (mmol/L), high-density lipoprotein (HDL) and low-density lipoprotein (LDL, mmol/L) cholesterol, triglycerides (mmol/L), erythrocyte sedimentation rate (ESR, mm/h), serum IgA antibodies against transglutaminase, serum IgA and serum levels of S-25-dihydroxyvitamin D (calcidiol) using routine laboratory protocols. Blood samples were also analysed for high-sensitivity C-reactive protein (hsCRP, mg/L), interleukin-1ß (IL-1ß), interleukin-1 receptor antagonist (IL-1RA), IL-6, IL-17, interferon-γ (IFN-γ), monocyte chemotactic protein (MCP) and TNF-α.

### Spinal mobility measurements and follow-up

Data on mobility measures was missing from one patient of the 66 patients participating in the original cross-sectional study, and from 11 after the cross-sectional study, leaving a total of 54 (41 males and 13 females) patients with complete data for further analysis. By using the recently published age-, sex- and length-specific reference intervals on spinal mobility of healthy individuals [4], a grading indicating the severity of the spinal immobility was calculated. Two points were given for a value lower than the 2.5th percentile and one point for a value lower than the 10th percentile of healthy individuals regarding 10-cm Schober test, lateral spinal flexion and cervical rotation respectively resulting in a grading ranging from 0 to 6. The spinal immobility grading from the last measurement available was used as the outcome.

### Statistical methods

To compare men and women, either an unpaired *t* test, chi-square or Mann-Whitney *U* test was used as appropriate. The associations between predictors and outcome, i.e. spinal immobility grading, were evaluated by linear regression. Multiple linear regression modelling, comprising variables selected on the basis of the univariate analyses and the scientific and clinical rationale, was used to model predictions of spinal immobility grading. Pearson’s correlation was used to assess associations between different predictors. Results were considered statistically significant at a two-tailed *p* value ≤ 0.05. Statistical calculations were performed with Stata for Macintosh version 13.1 (StataCorp, College Station, TX, USA).

## Results

### Patient characteristics

The clinical characteristics of the 54 patients in this follow-up are described in Table 1.

**Table 1:** Characteristics of 54 patients with ankylosing spondylitis (AS) participating in annual examinations of spinal immobility. Data are presented as mean (SD) unless otherwise stated

	Total (*n* = 54)	Men (*n* = 41)	Women (*n* = 13)	*p*
Age at end of follow-up (years)	54.7 (10.5)	53.4 (11.3)	58.6 (6.4)	0.13
Disease duration at the end of follow-up (years)	30.9 (11.7)	29.0 (11.8)	36.9 (9.0)	**0.03**
Duration of follow-up (years)	5.9 (2.3)	5.9 (2.3)	5.9 (2.1)	0.96
BASDAI	3.8 (1.7)	3.6 (1.5)	4.4 (2.1)	0.12
BASFI	2.7 (1.7)	2.4 (1.6)	3.6 (1.9)	**0.03**
ESR (mm/h)	15.6 (10.9)	14.7 (10.9)	18.5 (10.7)	0.28
hsCRP (mg/L)	4.2 (2.5)	4.6 (2.5)	2.7 (1.8)	**0.02**
Body mass index (kg/m^2^)	27.3 (5.2)	27.9 (5.6)	25.5 (3.4)	0.15
Smokers, *n* (%)	8 (14.8)	3 (7.3)	5 (38.5)	**0.006**
Past smokers, *n* (%)	17 (31.5)	13 (31.7)	4 (30.8)	**0.004**
NSAID, regularly, *n* (%)	35 (64.8)	27 (65.9)	8 (61.5)	0.78
NSAID, when needed, *n* (%)	13 (24.1)	9 (22.0)	4 (30.8)	0.52
DMARD, *n* (%)	6 (11.1)	5 (12.2)	1 (7.7)	0.77
Corticosteroids, *n* (%)	4 (7.4)	2 (4.9)	2 (15.4)	0.65
Exercise in leisure time, median (range)^a^	4 (1–5)	4 (2–5)	4 (1–5)	**0.04**
Physical activity at work, median (range)^b^	2 (1–4)	3 (1–4)	2 (1–4)	0.41

Bold font to indicate statistically significant result at *p*<0.05

*BASDAI*, Bath Ankylosing Spondylitis Disease Activity Index; *BASFI*, Bath Ankylosing Spondylitis Functional Index; *ESR*, erythrocyte sedimentation rate (Westergren); *hsCRP*, high-sensitivity C-reactive protein, *NSAID*, non-steroid anti-inflammatory drug; *DMARD*, disease-modifying anti-rheumatic drug

^a^Assessed by a five grade scale taking into account frequency, intensity and duration

^b^Assessed by a four grade scale taking into account frequency, intensity and duration

Women had longer disease duration and worse BASDAI and BASFI scores, but had lower hsCRP compared to the male patients. For the whole group, the mean (SD) of hsCRP was 4.2 (2.5) mg/L, with a min-max range of 0.2–8.4 mg/L. At the last available measurement, the patients had a mean disease duration of 30.9 (11.7) years with a mean follow-up time of 5.9 years (2.3). Eight patients had normal spinal mobility at the end of follow-up (above the 10th percentile of reference values for healthy populations for all of the included spinal measurements) despite a median disease duration of 27.9 years (range 11.7–49.0).

### Associations with spinal immobility

Less exercise during leisure time, higher levels of hsCRP, higher white blood cell count and platelet concentration (*p* = 0.019, *p* = 0.021, *p* = 0.026 and *p* = 0.024, respectively) were independently associated with a more pronounced spinal immobility grading at the end of follow-up (Table 2).

**Table 2:** Impact of covariates on the spinal immobility among 54 patients with ankylosing spondylitis after a mean follow-up time of 5.9 years. Univariate linear regression analyses

	Spinal immobility		
	*B*	95% CI	*p*
Disease duration	0.03	− 0.02 to 0.08	0.268
Physical activity at work	− 0.05	− 0.70 to 0.59	0.871
Exercise during leisure time	− 0.75	− 1.38 to − 0.13	**0.019**
BASDAI	− 0.24	− 0.58 to 0.09	0.146
BASFI	0.24	− 0.08 to 0.57	0.142
ESR	0.04	− 0.02 to 0.09	0.169
hsCRP	0.26	0.04 to 0.49	**0.021**
Body mass index	0.04	− 0.08 to 0.15	0.519
White blood cell count	0.29	0.04 to 0.54	**0.026**
Platelet concentration	0.01	0.00 to 0.02	**0.024**
Cholesterol	0.28	− 0.26 to 0.83	0.304
Serum triglycerides	0.63	− 0.42 to 1.68	0.236
HDL	− 0.10	− 1.40 to 1.20	0.873
LDL	0.33	− 0.34 to 1.01	0.323

Bold font to indicate statistically significant result at *p*<0.05

*PAL*, physical activity level; *BASDAI*, Bath Ankylosing Spondylitis Disease Activity Index; *BASFI*, Bath Ankylosing Spondylitis Functional Index; *ESR*, erythrocyte sedimentation rate (Westergren); *hsCRP*, high-sensitivity C-reactive protein; *HDL*, high-density lipoprotein; *LDL*, low-density lipoprotein

Disease activity, as assessed by BASDAI or ESR, was not associated with spinal immobility grading, neither was physical activity at work. No statistically significant associations were observed between levels of cytokines, vitamin D and transglutaminase and spinal immobility (data not shown). Neither were there any statistically significant differences comparing consumers and non-consumers of NSAIDs, DMARDs and corticosteroids (data not shown). Leucocyte count was associated with hsCRP (*p* = 0.007) and platelet concentration with ESR (*p* = 0.004).

In multiple regression models, disease duration, levels of hsCRP and exercise in leisure time could be attributed to approximately 25% of the spinal immobility grading (adjusted *r*-square 0.25; Table 3).

**Table 3:** Multiple linear regression models depicting predictors of spinal immobility at a mean of 5.9 years later among 54 patients with ankylosing spondylitis

Model^a^	1		2		3		4		5	
Variable	Coef. (95% CI)	*p*	Coef. (95% CI)	*p*	Coef. (95% CI)	*p*	Coef. (95% CI)	*p*	Coef. (95% CI)	*p*
Sex	− 0.23 (− 1.55 to 1.09)	0.731	− 0.02 (− 1.29 to 1.25)	0.972						
Disease duration at study end	0.07 (0.02 to 0.12)	**0.011**	0.07 (0.02 to 0.12)	**0.005**	0.07 (0.02 to 0.12)	**0.004**	0.08 (0.03 to 0.13)	**0.002**	0.08 (0.03 to 0.13)	**0.004**
hsCRP	0.39 (0.15 to 0.64)	**0.002**	0.36 (0.12 to 0.60)	**0.004**	0.36 (0.13 to 0.59)	**0.003**	0.37 (0.14 to 0.60)	**0.002**	0.35 (0.12 to 0.58)	**0.004**
Exercise in leisure time			− 0.72 (− 1.31 to − 0.12)	**0.019**	− 0.72 (− 1.30 to − 0.14)	**0.017**	− 0.74 (− 1.33 to − 0.16)	**0.014**	− 0.72 (− 1.31 to − 0.13)	**0.018**
NSAID							0.59 (− 0.48 to 1.66)	0.273	0.73 (− 0.40 to 1.86)	0.200
BASDAI									− 0.13 (− 0.45 to 0.19)	0.413
Adjusted *R*^2^	0.16		0.23		0.25		0.25		0.25	

Bold font to indicate statistically significant result at *p*<0.05

*hsCRP*, high-sensitivity C-reactive protein; *NSAID*, regular consumption of non-steroid anti-inflammatory drug; *BASDAI*, Bath Ankylosing Spondylitis Disease Activity Index

^a^Regression constant not shown

## Discussion

In the present study, disease duration, exercise habits and inflammation, as measured by hsCRP, were associated with subsequent development of spinal immobility in AS in multiple regression models. Adding sex, BASDAI or NSAID consumption to the analysis did not improve the models. In clinical experience among rheumatologists and health professionals, as well as among patients, an active life style with physical activity and regular exercise improves the long-term prognosis of AS. Intervention studies have shown that exercise can have a positive effect on pain and disease activity, but long-term studies evaluating spinal immobility are scarce [10]. Exercise has long been an important part of the patients’ self-care programmes, and the possibility of a long-term effect on the mobility parallel to a number of other positive effects, such as decreasing fatigue [11], improving sleep [12] and decreasing the risk for numerous chronic diseases [13], can be an extra motivation to exercise. Beside exercise, this study identified hsCRP and disease duration as predictors for spinal immobility. Due to the nature of AS being chronic and progressive, duration of the disease might be an expected risk factor for the development of stiffness. High-sensitivity C-reactive protein has previously been described to be associated with radiographic progression [14, 15]. The hsCRP concentrations in the present study were overall normal, or slightly elevated, with the mean of 4.2 mg/L and a maximal value of 8.4 mg/L. This indicates that also low-grade systemic inflammation may have a negative impact on the long-term prognosis regarding spinal immobility.

The findings that BASDAI did not influence future impairment of spinal mobility might be unexpected, but in our clinical experience, it is not uncommon that patients with a verified diagnosis of AS do not develop spinal immobility despite extensive issues of pain. Furthermore, an advanced bamboo spine may occasionally be discovered by coincidence on radiological examinations performed for other reasons, e.g. a chest X-ray, or a computerised tomography due to a trauma in a previously undiagnosed patient who never was particularly affected by spinal pain. The development of structural changes may not necessarily be associated with prominent pain, and vice versa.

The early onset and the slow progression of the AS disease make evaluation of the prognosis a challenge using traditional cohort or case-control designs [16, 17]. To evaluate the development of spinal immobility, patients must be followed over a long time, during which time treatment guidelines as well as societal changes influencing the life style habits will change, i.e. during the years needed to develop spinal immobility, the pharmacological treatment for each patient will vary, as well as modifiable risk factors such as exercise or smoking habits.

The main strength in this study is the well-defined and representative population of AS patients followed over a long time. The main limitation is the limited sample size, mainly inherited from the previously performed cross-sectional study. The patient cohort is heterogeneous with regard to disease duration and follow-up time between the first and last spinal measurement. Similar measurements in a more homogenous population with standardised time points between measurements would probably perform better. It is notable that, due to the original study protocol in the cross-sectional study, patients on biological treatment were excluded [6] However, since only 14% of the patients were treated with biologics at the time of the study [18], the risk for any selection bias towards less affected patients in the study is small. Although we had extensive data on spinal mobility, some data, such as measurement of intermalleolar distance and patient’s assessment of global health, was lacking, rendering it not possible to use common instruments such as BASMI and ASDAS in the analyses. Nonetheless, by using the previously collected clinical and research data together with published reference intervals of spinal immobility in a new context, we could point out exercise as a modifiable risk factor and low-grade inflammation, as assessed by hsCRP, as a marker for subsequent development of spinal immobility in AS, and these results adhere to other studies as well as to clinical experience.

To conclude, in the present longitudinal study on AS patients with established disease, we found disease duration, exercise habits and hsCRP to be associated with the development of spinal immobility. This highlights that exercise, as a modifiable risk factor, should remain an important part of the non-pharmacological treatment and self-care for patients with AS, with a range of possible positive effects, including preserved spinal mobility. The study also implicates that optimal control of systemic inflammation may impact the prognosis regarding spinal immobility in the long term.
